# Microbial Degradation Behavior in Seawater of Polyester Blends Containing Poly(3-hydroxybutyrate-*co*-3-hydroxyhexanoate) (PHBHHx)

**DOI:** 10.3390/md16010034

**Published:** 2018-01-17

**Authors:** Hitoshi Sashiwa, Ryuji Fukuda, Tetsuo Okura, Shunsuke Sato, Atsuyoshi Nakayama

**Affiliations:** 1 BDP Group, New Buisiness Development Department, Kaneka Co., 5-1-1, Torikai-Nishi, Settsu, Osaka 566-0072, Japan; Ryuji.Fukuda@kaneka.co.jp; 2 BDP Processing Technology Development Team, Plastics Molding & Processing Technology Development Group, Kaneka Co., 5-1-1, Torikai-Nishi, Settsu, Osaka 566-0072, Japan; Tetsuo.Okura@kaneka.co.jp; 3 BDP Group, Biotechnology Development Laboratories, Kaneka Co., 8-1 Miyamae-cho, Takasago, Hyogo 676-8688, Japan; Shunsuke.Sato@kaneka.co.jp; 4 Biomolecule Design Research Group, Biomedical Research Institute, National Institute of Advanced Industrial Science and Technology (AIST), 1-8-31, Midorigaoka, Ikeda, Osaka 563-8577, Japan; a.nakayama@aist.go.jp

**Keywords:** poly(3-hydroxybutyrate-*co*-3-hydroxyhexanoate) (PHBHHx), blendsurface morphology, microbial, degradation, seawater

## Abstract

The microbial degradation behavior of poly(3-hydroxybutyrate-*co*-3-hydroxyhexanoate) (PHBHHx) and its compound with several polyesters such as poly(butylene adipate-*co*-telephtharate) (PBAT), poly(butylene succinate) (PBS), and polylactic acid (PLA) in seawater was tested by a biological oxygen demand (BOD) method. PHBHHx showed excellent biodegradation in seawater in this study. In addition, the biodegradation rate of several blends was much influenced by the weight ratio of PHBHHx in their blends and decreased in accordance with the decrement of PHBHHX ratio. The surface morphology of the sheet was important factor for controlling the biodegradation rate of PHBHHx-containing blends in seawater.

## 1. Introduction

In recent years, there is increasing interest in marine pollution owing to the effect of microplastics [1,2]. Microplastics are defined as particles of less than 5 mm in size and depending on the plastic, it has been shown that various chemicals and toxic blends can be absorbed onto their surface [3]. For this, it is great concern that these blends could be accumulated into zooplankton, fish, and finally to mammals through the food chain. Therefore, the dynamics of production, distribution, diffusion, and degradation of microplastics in marine environments are receiving a lot of attention.

**Figure 1:**
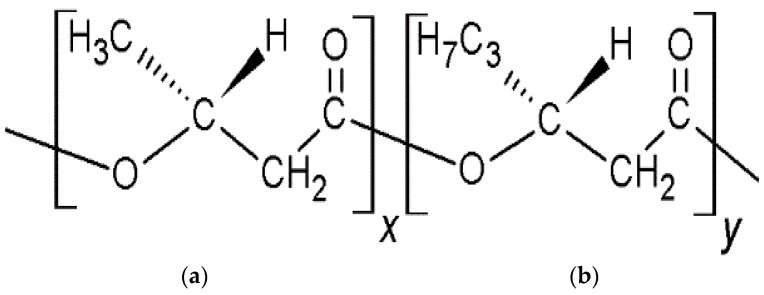
Chemical structure of poly(3-hydroxybutyrate-*co*-3-hydroxyhexanoate) (PHBHHx). (**a**) hydroxybutyrate (3-HB) unit; (**b**) 3-hydroxyhexanoate (3-HHx) unit.

One of the possible solutions toward this problem is focused on the application of biodegradable plastics. Polyhydroxyalkanoates (PHAs) have the most promising biodegradable properties amongst the class of several biodegradable plastics [4,5]. Since PHA plastics degrade and disappear in seawater, toxic blends bound microplastics have no existence in marine environment [6]. Among the PHAs, poly(3-hydroxybutyrate-*co*-3-hydroxyhexanoate) (PHBHHx, Figure 1) shows excellent flexibility, because the 3-hydroxyhexanoate (3-HHx) unit (Figure 1b) is not taken into the crystallized 3-hydroxybutyrate (3-HB) unit (Figure 1a) in the crystal structure. Therefore, the crystallinity of this polymer can be controlled by changing the percentage of the 3-HHx unit [7].

PHAs including PHBHHx show good biodegradation properties in soil, as for other biodegradable polyesters such as poly(butylene adipate-*co*-telephtharate) (PBAT), poly(butylene succinate) (PBS), poly(butylene succinate-*co*-adipate) (PBSA), polycaprolactone (PCL), etc. [8,9,10]. Especially, it has been reported that PHAs such as PHB and poly(3-hydroxybutyrate-*co*-valerate) (PHBV) or PCL showed excellent biodegradation in seawater [11,12,13,14,15,16]. By contrast, polylactic acid (PLA), which is one of the most utilized biodegradable polyesters, shows poor degradability under ambient conditions, although it is possible to degrade under industrial composting conditions [17]. On the other hand, PHAs are polyesters biologically synthesized by various microorganisms from renewable resources such as saccharides, vegetable oils, glycerol, and others. PHA-degrading bacteria have been isolated from most environments and produce enzymes (extracellular PHA depolymerase) to degrade PHAs under both anaerobic and aerobic conditions. In general, PHAs include PHBHHx showed, blends with other polyesters such as PBAT, PLA, and PBS were carried out to make up the mechanical properties [10]. Therefore, the biodegradability of these blends in seawater needs to be investigated.

In this study, we investigated the effect on seawater biodegradability of different highly biodegradable (as PBAT and PBS) and poorly biodegradable (as PLA) polyesters in the blends (powder and sheets) with PHBHHx at different ratios. Because PHBHHx shows excellent flexibility and heat resistant properties, PHBHHx is used in not only its independent form but also as blends with several biodegradable polyesters. Moreover, the effect of surface morphology of PHBHHx/PBAT and PHBHHx/PLA sheets was also investigated in this study.

## 2. Results and Discussion

### 2.1. Biodegradation of PHBHHx and PHBHHx/PBAT Blends

In this study, 11 mol% of 3-HHx unit content of PHBHHx was used. Table 1 shows the bending elastic modulus and the molecular weight of polymers used in this study before and after biodegradation in seawater for 28 days. PBAT, PBS, and PLA, which were not shown microbial metabolism estimated by biological oxygen demand (BOD) test, showed slight decrease of molecular weight by hydrolysis in seawater. On the other hand, PHBHHx, which showed microbial metabolism, did not show molecular weight change. This phenomenon could be explained as follows: first the surface of PHBHHx was hydrolyzed by microbial enzymes, and then seawater soluble oligomers of PHBHHx were metabolized by microorganisms in seawater. Since it has been shown that PHBHHx showed high resistance of hydrolysis in comparison with those of PBAT, PBS, and PLA, the remained PBHHx would still keep the high molecular weight.

**Figure 2:**
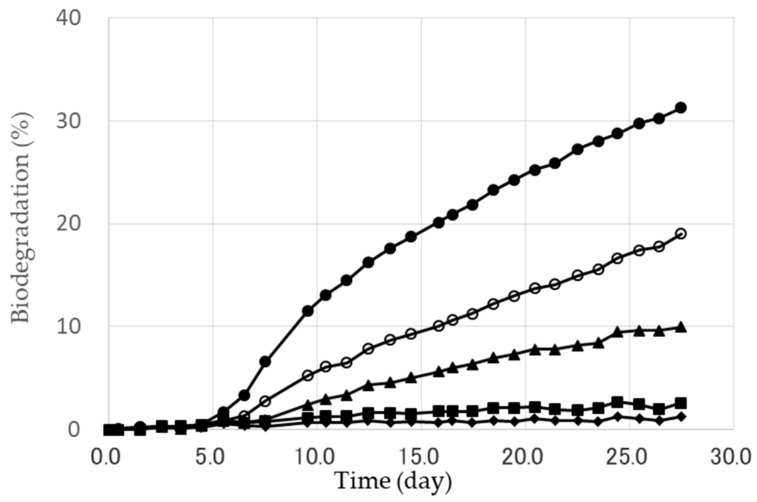
The biodegradation of PHBHHx and PHBHHx/PBAT blends (powder shape) in seawater. ●: PHBHHx, ○: PHBHHx/PBAT = 80/20 (wt/wt), ▲: PHBHHx/PBAT = 60/40 (wt/wt), ■: PHBHHx/PBAT = 40/60 (wt/wt), ♦: PBAT.

Figure 2 shows the typical time courses of the biodegradation of PHBHHx and PHBHHx/PBAT blends (powder form) in seawater as indicated by their biological oxygen demand (BOD). The biodegradation of PHBHHx gradually increased with the degradation time. The pure PHBHHx showed excellent biodegradability in seawater in comparison with the original PBAT, whose biodegradability was quite low level. The degree of biodegradation therefore showed much dependence on the effect of the weight ratio in the blend. The biodegradation was decreased with the ratio of PHBHHx. 

The biodegradation degree (%) in seawater after 28 days is summarized in Table 2. The particle size distribution of all powder form of samples were around 24–2000 μm. 

To confirm only the PHBHHx component degrade in the blends, the content (wt %) of PHBHHx in the blends were analyzed by ^1^H-NMR analysis before and after biodegradation (Figure S1). The contents of PHBHHx before biodegradation were a little bit changed after the melt exclusion process. As shown in Table 2, the contents of PHBHHx were slightly decreased after biodegradation, which means that PHBHHx component surely degraded with seawater.

### 2.2. Biodegradation of PHBHHx/PBS Blends

The biodegradation degree (%) of PHBHHx/PBS blends in seawater after 28 days is summarized in Table 3. In the case of PHBHHx/PBS, the biodegradability also showed much dependence on the PBS ratio in the blends. On comparing with the relative rates (calcd. and found), PHBHHx/PBS = 80/20 (entry 2) was not found to inhibit the biodegradation rate. However, the 60/40 and 40/60 blends (entries 3 and 4) showed the same level of inhibition as the PBAT blends. These results suggest that a high amount of PBS also inhibits the biodegradability of PHBHHx. In these cases, PHBHHx components were surely decreased after biodegradation. The content (wt %) of PHBHHx in the blends were analyzed by ^1^H-NMR analysis before and after biodegradation (Figure S3). And Figure S4 shows the time courses of the biodegradation of PHBHHx/PLA blends (powder form) in seawater.

### 2.3. Biodegradation of PHBHHx/PLA Blends

**Figure 3:**
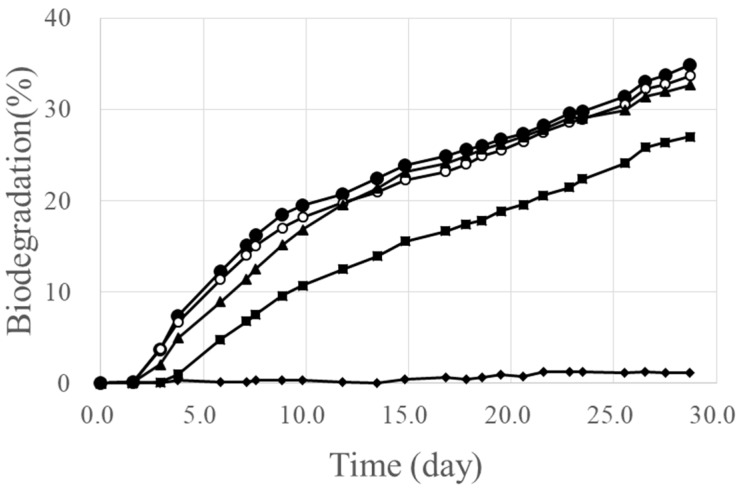
The biodegradation of PHBHHx and PHBHHx/PLA blends (powder shape) in seawater. ●: PHBHHx, ○: PHBHHx/PLA = 80/20 (wt/wt), ▲: PHBHHx/PLA = 60/40 (wt/wt), ■: PHBHHx/PLA = 40/60 (wt/wt), ♦: PLA.

Figure 3 shows the time courses of the biodegradation of PHBHHx/PLA blends (powder form) in seawater. The original PLA showed quite low biodegradability in seawater, as for the PBAT and PBS. However the biodegradation of PHBHHx/PLA blends showed less dependence on the PLA ratio. The biodegradation degree (%) in seawater after 28 days is summarized in Table 4. In these cases, PHBHHx components were surely decreased after biodegradation. The reason of relatively high biodegradation the PHBHHx/PLA blends of 80/20, 60/40, and 40/60 is discussed later (Section 2.5). The content (wt %) of PHBHHx in the blends were analyzed by ^1^H-NMR analysis before and after biodegradation (Figures S2-1 and S2-2). And Figure S5 shows the particle size distribution of PHBHHx/PLA blend powders.

### 2.4. Effect of the Morphology of PHBHHx/PBAT Compound on the Biodegradation

**Figure 4:**
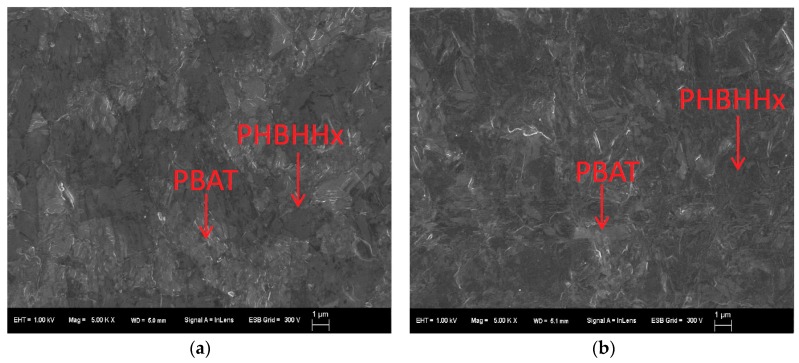
Typical scanning electron microscope (SEM) micrographs of the blend of PHBHHx/PBAT = 80/20 (**a**) and 60/40 (**b**) sheet surface (×5000) before biodegradation. The scale bar length is 1 μm.

Table 5 shows the effect of the form of the PHBHHx/PBAT blends on the biodegradation rate in seawater after 28 days. In the most cases, the sheet forms (entries 2 and 4) showed less biodegradability in comparison with those of powder form. To clarify this reason for these difference, transmission electron microscope (TEM) and scanning electron microscope (SEM) analyses were performed. Figure 4 shows the SEM image of the RuO4 stained PHBHHx/PBAT = 80/20 and 60/40 blend sheets surface before biodegradation. The PBAT was the brighter part owing to a higher scattering intensity of the electron beam by the attachment of RuO_4_ to the phenyl group in PBAT compared with the PHBHHx component. Despite the high PHBHHx fraction in PHBHHx/PBAT = 80/20 and 60/40, a large proportion of the PBAT component was observed at the surface of these sheets, which would be caused by the lower molecular weight of PBAT layer tends to exposure at the surface area under preparing T-die sheet. This would explain the decrease of biodegradation for the PBAT blends because the PBAT at the surface restricted microorganial access to PHBHHx.

**Figure 5:**
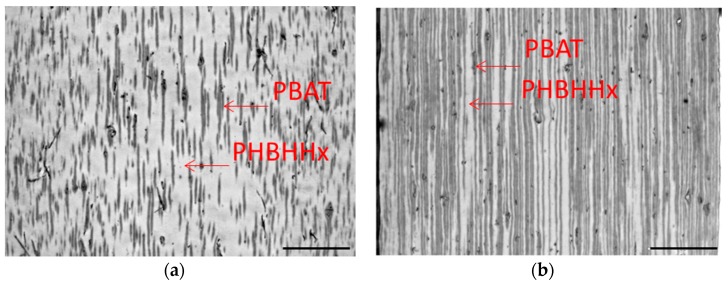
Typical transmission electron microscope (TEM) micrographs of the cross section of a sheet of PHBHHx/PBAT = 80/20 (**a**) and 60/40 (**b**) before biodegradation. The scale bar length is 5 μm.

Figure 5 shows the TEM photograph of a cross section of the PHBHHx/PBAT = 80/20 and 60/40 sheets before biodegradation. The PBAT component was darks owing to less permeability of electron beam through the attachment of RuO_4_ to the phenyl group in the PBAT compared with the PHBHHx part. The PBAT layer structure (black parts) was arranged like an annual ring of tree for the 60/40 blend in comparison with that of 80/20 which was more irregular. This difference means that the biodegradation was more inhibited for the 60/40 blend. Since the PBAT did not show biodegradation, the biodegradation of PHBHHx/PBAT blends was highly inhibited at the PBAT layer. Overall, these morphological effects cause the decrease of biodegradability in PHBHHx/PBAT sheet.

### 2.5. Effect of the Morphology of PHBHHx/PLA Compound on the Biodegradation

Table 6 shows the effect of the form of the PHBHHx/PLA blends on their biodegradation in seawater after 28 days. In these cases, the sheet forms (entries 4 and 6) also showed less biodegradability in comparison with those in powder forms. In the previous paper, the PLA/PHA biodegradable blends for pneumothermic fablication of nonwoven was reported [18]. However, there is no information on the morphology of PHA and PLA blends. To clarify the reason for this, scanning electron microscope (SEM) analysis were performed.

**Figure 6:**
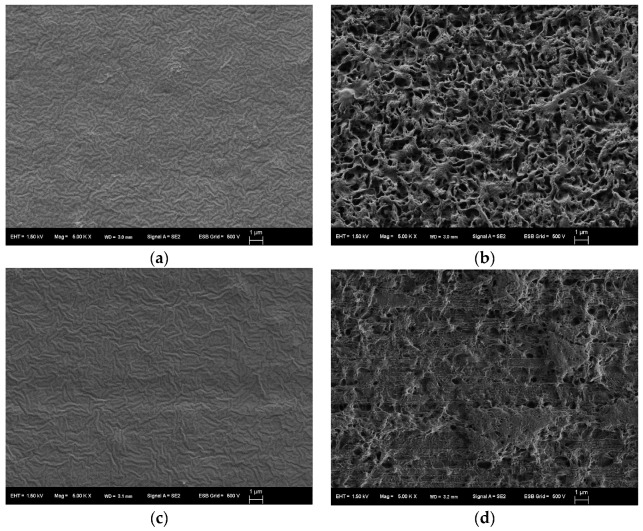
SEM photograph (×5000) of the blend sheets of (**a**) PHBHHx/PLA = 80/20 before, (**b**) PHBHHx/PLA = 80/20 after, (**c**) PHBHHx/PLA = 60/40 before, (**d**) PHBHHx/PLA = 60/40 after biodegradation in seawater. The scale bar length is 1 μm.

Figure 6 shows the typical SEM photograph of the sheet surface of the PHBHHx/PLA 80/20 blend before and after biodegradation in seawater. A lot of hollows were observed on the surface of this highly biodegradable sheet after biodegradation (Figure 6b). By contrast, the less biodegradable sheet (60/40) had a relatively flat structure after seawater exposure (Figure 6d). This suggests that the surface of the less degradable sheet (60/40) was more covered with the low-degradable PLA component than PHBHHx component. Therefore, the masking effect of these blend sheets could be the reason for less biodegradability. In conclusion, the surface morphology of the melt blend sheets was an important factor for controlling the biodegradation in seawater.

**Figure 7:**
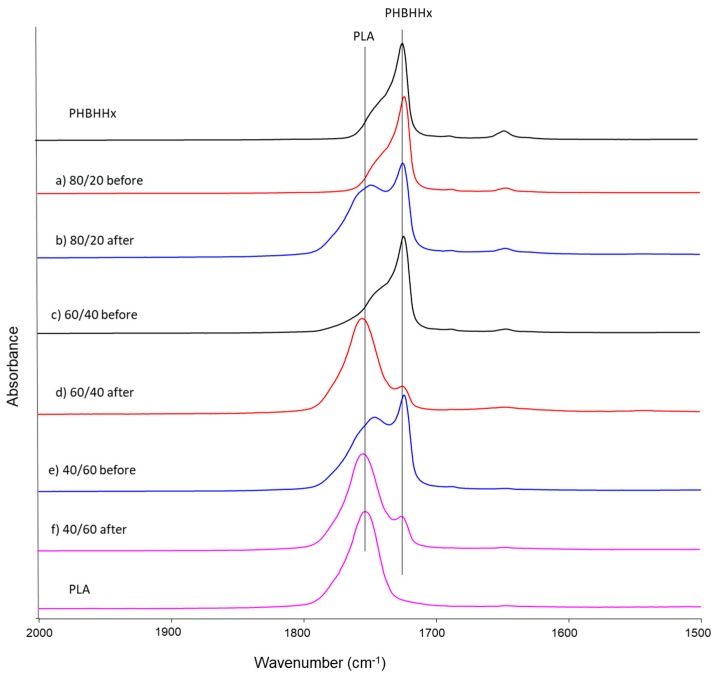
Fourier-transform infrared spectroscopy (FT-IR) by attenuated total reflectance (ATR) spectra of sheets of PHBHHx, PLA, and their blends before and after biodegradation in seawater.

Figure 7 shows the surface the fourier-transform infrared spectroscopy (FT-IR) by attenuated total reflectance (ATR) spectroscopy of sheets of PHBHHx, PLA, and their blends before and after 28 days of biodegradation in seawater. The peak areas of the C=O stretching bands at 1756 cm^−1^ corresponding to the PLA component and those at 1721 cm^−1^ corresponding to the PHBHHx component were compared before and after biodegradation. As shown in Figure 7, the peak area corresponding to the PHBHHx decreased and the PLA area increased after biodegradation in seawater. In the case of PHBHHx/PLA = 60/40 and 40/60, especially, a significant change was observed in Figure 7c–f. This suggests that the ratio of PLA components increased at the surface of the blend sheets after biodegradation, indicating that the PHBHHx components degrade preferentially on the surface of PHBHHx/PLA blend sheets. As shown in Table 1, PLA shows higher bending elastic modulus than that of PHBHHx. Therefore, the reason of high biodegradation the PHBHHx/PLA blends powders would be caused by the increment of surface of PHBHHx, which is formed some clacking at the freeze pulverization process. These increment of the surface area of PHBHHx would be supported by the clear decrease of surface PHBHHx component by FT-IR spectroscopic analyses.

## 3. Materials and Methods

### 3.1. Materials

A commercial grade of PHBHHx (3-HHx unit content = 11 mol%, X151A, ©KANEKA Biodegradablepolymer™ PHBH, Kaneka Co., Osaka, Japan) was used in this study. PBAT (Ecoflex C-1200, BASF), PLA (Ingeo 10361D, Nature Works, Minnetonka, MN, USA), and PBS (Bionolle 1020MD, Showa Denko, Tokyo, Japan) were used as obtained. Each blend (PHBHHx/PBAT, PHBHHx/PLA, PHBHHx/PBS) was prepared by a melt extrusion process using TEM26SS (Toshiba Machine, Numazu, Japan) at 140 °C with 80 rpm for 2 min to obtain each blended pellets. Each blended pellets were pulverized by freeze pulverization using a SPEX SamplePre 6750 Freezer/Mill (SPEX) to obtain blend powders. The average particle size of each blend powder was measured using a Microtruc MT3300EXII (Nikkiso, Tokyo, Japan). Sheets of the blends at 20 μm of thickness were also prepared by a T-die cast extrusion process at 140 °C for 8 min using a Labo-Plastmill Model 30C150 (Toyoseiki, Nagano, Japan). The data of each bending elastic modulus was cited from the previous study [10].

### 3.2. Biodegradation of Polyesters

Biodegradation of polyesters with seawater was evaluated using a BOD tester 200 F (TAITEC Co., Tokyo, Japan) according to the previous report [19]. Seawater was collected at the Osaka-Nanko bay area. The typical procedure is as follows: Seawater (200 mL), and sample (30 mg) were placed in a fermenter at 27 °C. Ca(OH)_2_ powder (1.0 g) was used as a trap for produced CO_2_ gas. At the prescribed time, the amount of consumed O_2_ gas was measured. The fermenter, which did not include the sample, was used as a control. The net amount of consumed O_2_ gas (mL) was evaluated as the difference of the sample and control. The theoretical amount of consumed O_2_ (mL) means the total O_2_ that should be consumed on complete degradation of sample. Biodegradation (%) was calculated as follows:

In the case of PHBHHx (11 mol% of 3-HHx content), the consumption of O_2_ is the following Equation (1), and the biodegradation is estimated by the Equation (2):

C_4.22_H_6.44_O_2_ + 4.83 O_2_ → 4.22 CO_2_ + 3.22 H_2_O, (1)

Biodegradation (%) = [experimentally consumed O_2_ (mL)/theoretical O_2_ (mL)] × 100. (2)

The consumption of O_2_ (mL) without samples was deleted from the experimentally consumed O_2_ (mL). The biodegradation value (%) was average value of duplicate data (*N* = 2). All biodegradation data using a BOD tester occurred by the microbial metabolism. Therefore, deionized water without microorganisms does not occur with the O_2_ consumption.

### 3.3. TEM and SEM Analysis

Transmission electron microscope (TEM) observation of each samples were carried out after treatment with RuO4 using a Hitachi H-7650 transmission electron microscope (Hitachi High-Technologies, Tokyo, Japan). Scanning electron microscope (SEM) observation of each samples were also carried out after treatment with RuO4 using Zeiss ULTRAplus scanning electron microscope (Zeiss, Oberkochen, Germany).

### 3.4. FT-IR Analysis

FT-IR spectroscopy of the sample surface by attenuated total reflectance (ATR) spectra was measured using a Perkin Elmer Co., Ltd. (Waltham, MA, USA) model Frontier FT-IR spectrometer.

### 3.5. ^1^H-NMR Analysis

^1^H-NMR analysis of each blends before and after biodegradation was measured using Brucker 400 MHx NMR spectrometer (Bruker Biospin Co., Yokohama, Japan) in CDCl_3_ as solvent. The content of PHBHHx, PBAT, and PHB was estimated from the following peak areas; PHBHHx = 5.27 ppm (CH), PLA = 5.17 ppm (CH), PBAT = 8.12 ppm (C_6_H_4_ of aromatic protons), PBS = 2.64 ppm (CH_2_ of succinate).

### 3.6. Molecular Weight Analysis

The molecular weight (Mn, Mw) of PHBHHx, PBAT, PLA, and PBS was determined by GPC (apparatus, Shimadzu LC-10A, Shimadzu Co., Kyoto, Japan, column, Shodex GPCK-806M, Showa Denko, Tokyo, Japan, eluent, chloroform, injection, 10 μL, column temperature, 40 °C) using polystyrene as standard at the concentration of 1.5 mg/mL.

## 4. Conclusions

The PHBHHx sheets and powders showed excellent biodegradability in seawater. The biodegradation of the blends of PHBHHx/PBAT, PHBHHx/PLA, and PHBHHx/PBS was significantly influenced by the weight ratio of their melt blends and decreased in accordance with the decrement of PHBHHx ratio from the BOD tester. The surface morphology of the PHBHHx/PBAT and PHBHHx/PLA blends sheet was an important factor for controlling the biodegradation in seawater based on the SEM and TEM analysis. The PHBHHx component at the surface decreased on degradation in seawater based on the FT-IR (ATR) analysis. From these results, it is clear that the PHBHHx component degraded across a range of blend compositions in seawater.

In this study, PHBHHx was shown to have excellent biodegradability in seawater, but the biodegradation rate of other biodegradable polymers was shown to be much lower than that of the PHBHHx. From the results of this study, PHBHHx-containing blends degrade much faster than conventional plastics like PBAT, PBS, and PLA and so on, indicating that PHA blends including PHBHHx are useful materials for the marine environment.

## Figures and Tables

**Table 1:** The molecular weight of polymers (sheet shape) used in this study before and after biodegradation in seawater for 28 days.

Polymer	Bending Elastic Modulus	Before Biodegradation			After 28 Days Biodegradation		
	MPa	M_n_	Mw	Mw/M_n_	M_n_	Mw	Mw/M_n_
PHBHHx	850	280,000	550,000	2.0	280,000	530,000	1.9
PBAT	110	63,000	120,000	1.9	55,000	110,000	2.0
PBS	650	81,000	150,000	1.9	77,000	126,000	1.6
PLA	3190	100,000	160,000	1.6	94,000	140,000	1.5

**Table 2:** The biodegradation degree (%) of PHBHHx/PBAT blends in seawater after 28 days.

Entry	PHBHHx/PBAT Added (wt/wt)	Form	Average Particle Size (μm)	Biodegradation (%)	Content of PHBHHx in Blends (wt %)	
					Before	After
1	100/0	Powder	440	31	- ^(a)^	-
2	80/20	Powder	380	19	83	80
3	60/40	Powder	400	10	66	61
4	40/60	Powder	400	3	47	46
5	0/100	Powder	470	1	-	-

^(a)^ Not tested.

**Table 3:** The effect of mixture ratio of PHBHHx/PBS blends on the biodegradation in seawater after 28 days.

Entry	PHBHHx/PBS (wt/wt)	Form	Average Particle Size (μm)	Biodegradation (%)	Content of PHBHHx in Blends (wt %)	
					Before	After
1	100/0	Powder	440	51	- ^(a)^	-
2	80/20	Powder	370	41	80	76
3	60/40	Powder	420	18	61	57
4	40/60	Powder	390	5	42	38
5	0/100	Powder	310	1	-	-

^(a)^ Not tested.

**Table 4:** The effect of mixture ratio of PHBHHx/PLA blends on the biodegradation in seawater after 28 days.

Entry	PHBHHx/PLA (wt/wt)	Form	Average Particle Size (μm)	Biodegradation (%)	Content of PHBHHx in Blends (wt %)	
					Before	After
1	100/0	Powder	440	34	- ^(a)^	-
2	80/20	Powder	420	33	82	77
3	60/40	Powder	430	32	64	62
4	40/60	Powder	440	26	51	45
5	0/100	Powder	490	1	-	-

^(a)^ Not tested.

**Table 5:** The effect of morphology of PHBHHx/PBAT blends on the biodegradation in seawater after 28 days.

Entry	Mixture	Ratio	Form ^(^^a)^	Biodegradation (%)	Effect of Morphology
1	PHBHHx/PBAT	80/20	Powder	19	
2		80/20	Sheet	10	○ ^(^^b)^
3		60/40	Powder	10	
4		60/40	Sheet	2	○
5		40/60	Powder	3	
6		40/60	Sheet	1	Non degradable

^(a)^ The thickness of each sheets is 20 μm; ^(b)^ ○, effect of morphology was observed.

**Table 6:** The effect of morphology of PHBHHx/PLA blends on the biodegradation in seawater after 28 days.

Entry	Mixture	Ratio	Form ^(^^a)^	Biodegradation (%)	Effect of Morphology
1	PHBHHx/PLA	80/20	Powder	33	
2		80/20	Sheet	42	None
3		60/40	Powder	32	
4		60/40	Sheet	20	None
5		40/60	Powder	26	
6		40/60	Sheet	8	○ ^(b)^

^(a)^ The thickness of each sheets is 20 μm; ^(b)^ ○, effect of morphology was observed.

## Supplementary Materials

The following are available online at www.mdpi.com/1660-3397/16/1/34/s1, Figure S1: ^1^H-NMR spectra of PHBHHx/PBAT = 80/20, Figure S2-1: ^1^H-NMR spectra of PHBHHx/PLA = 80/20, Figure S2-2: Enlarged view of the ^1^H-NMR spectra of PHBHHx/PLA = 80/20, Figure S3: ^1^H-NMR spectra of PHBHHx/PBS = 80/20, Figure S4: The biodegradation of PHBHHx and PHBHHx/PBS blends (powder shape) in seawater, Figure S5: Particle size distribution of PHBHHx/PLA blend powders.
