# Supplementary material for: Microbial Degradation Behavior in Seawater of Polyester Blends Containing Poly(3-hydroxybutyrate-co-3-hydroxyhexanoate) (PHBHHx)
Source: Mar Drugs. 2018 Jan 17;16(1):34. doi: 10.3390/md16010034 (PMC5793082; doi:10.3390/md16010034)
Supplement: Supplementary file 1 [file marinedrugs-16-00034-s001.pdf]

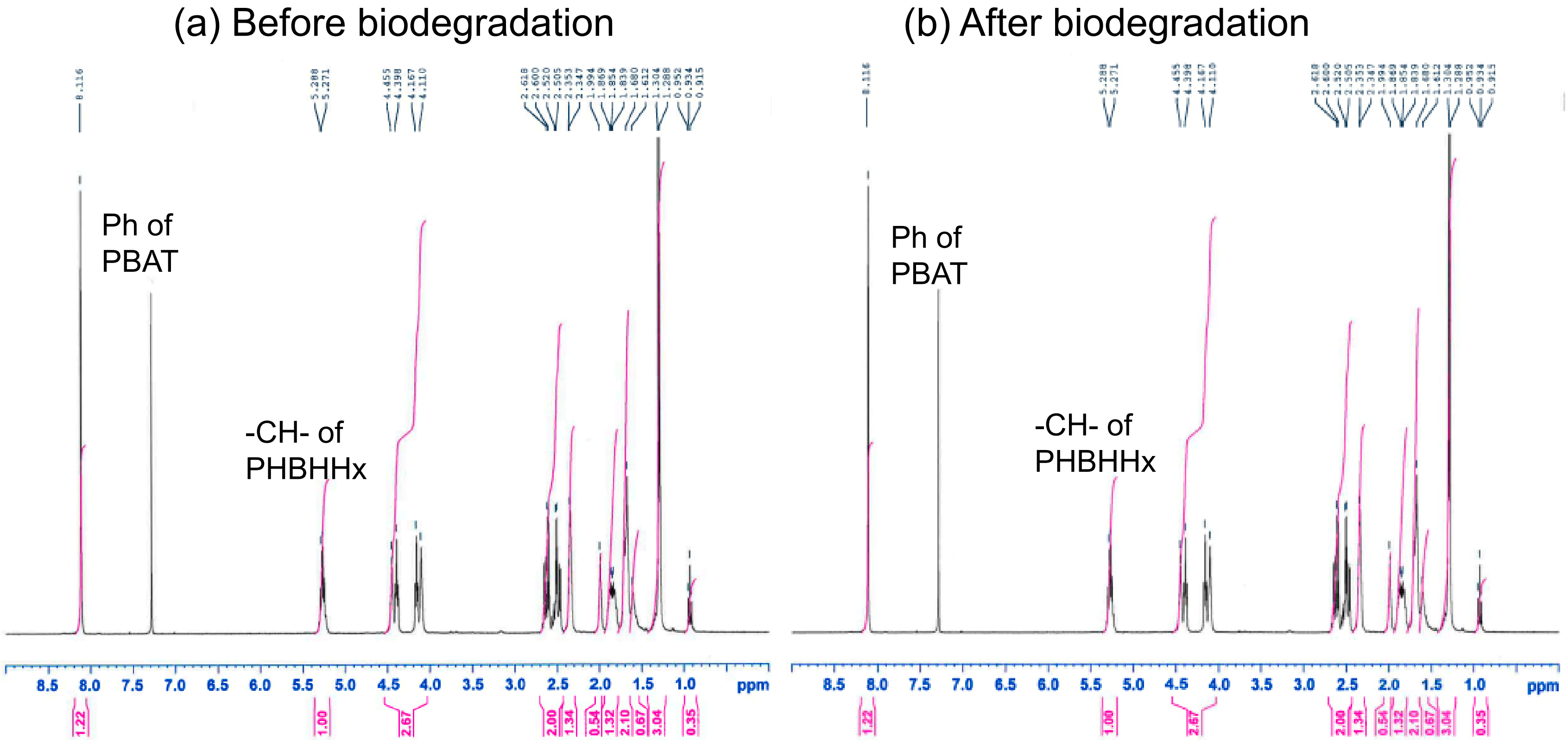

Figure S1. ^{1}H NMR spectra of PHBHHx/PBAT=80/20 (a) before and (b) after biodegradation.

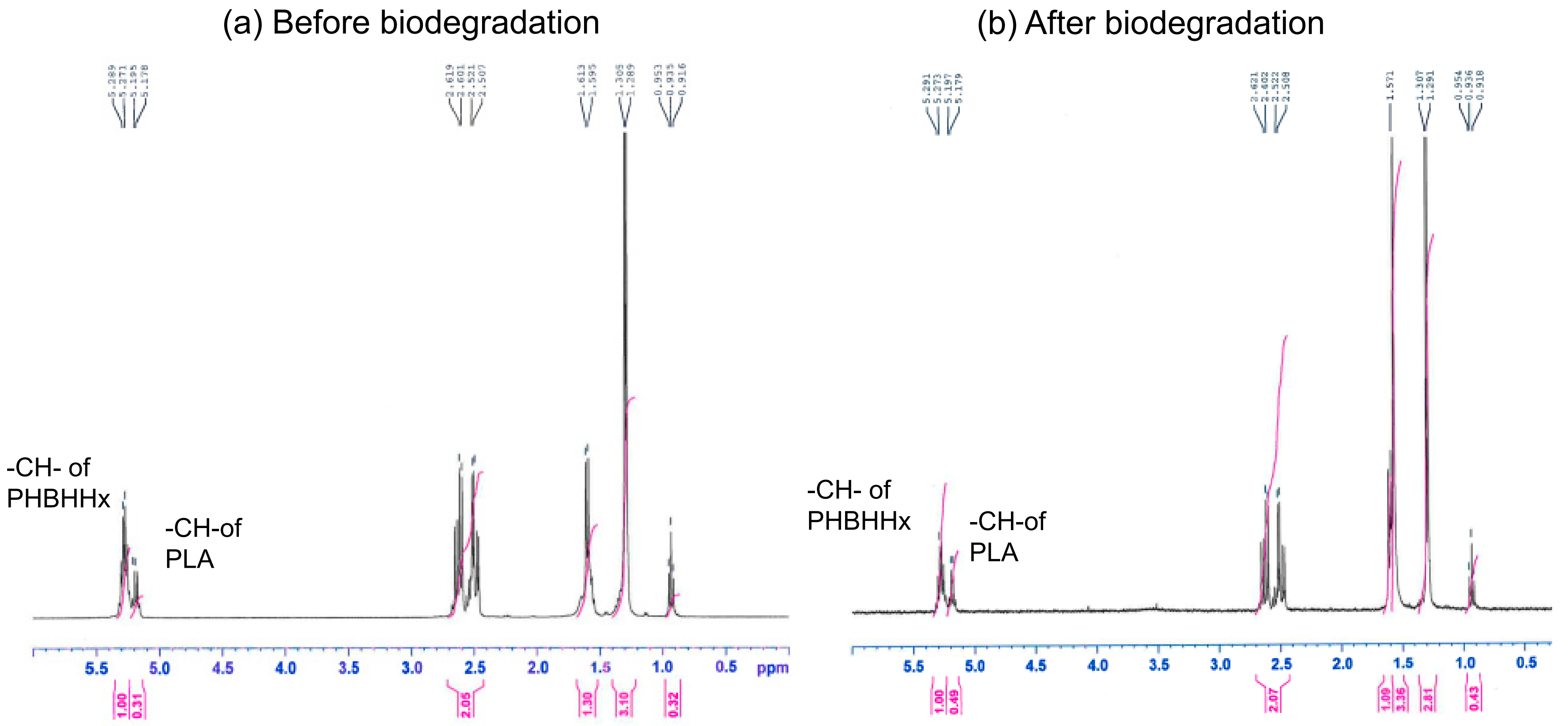

Figure S2-1. ^{1}H NMR spectra of PHBHHx/PLA=80/20 (a) before and (b) after biodegradation.

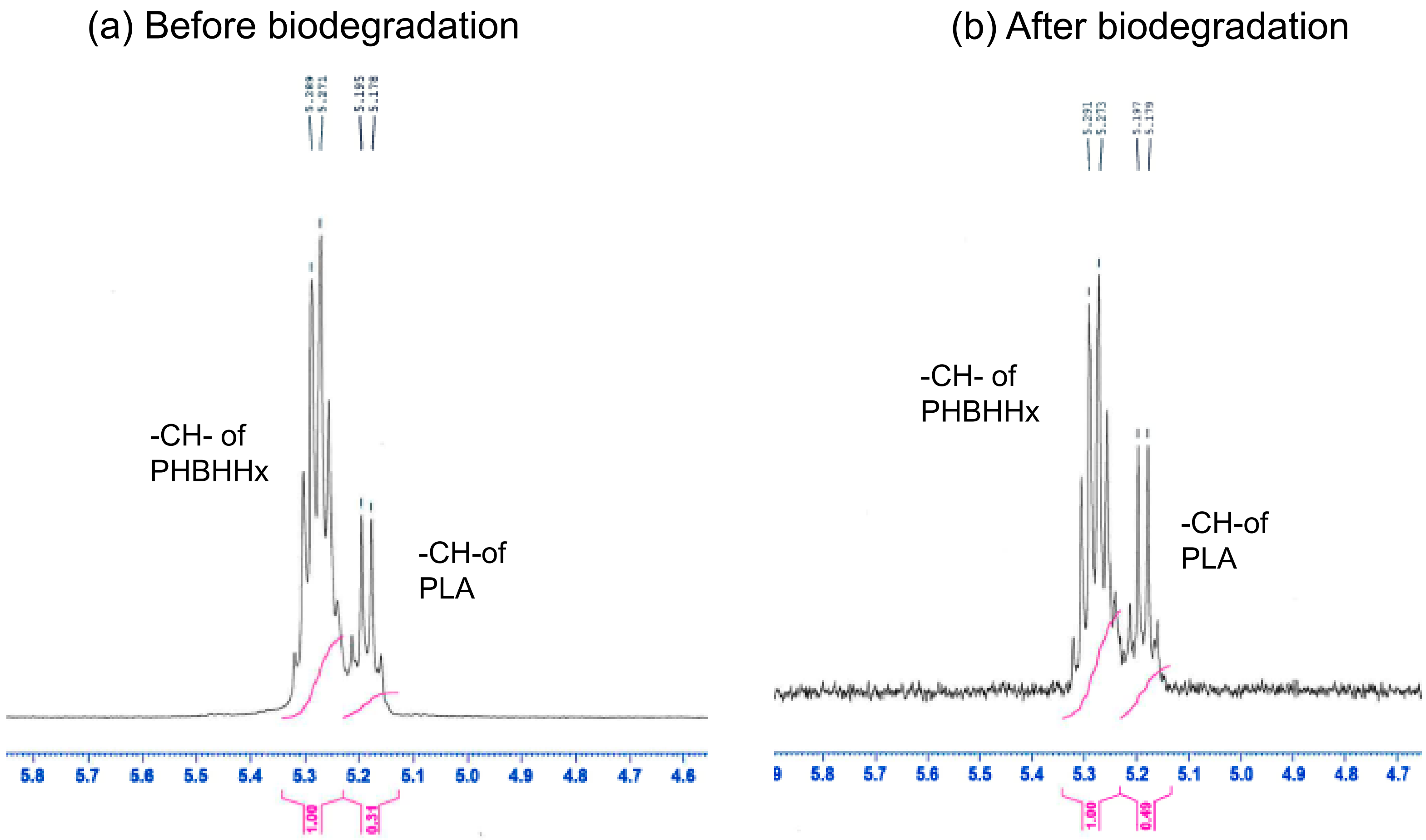

Figure S2-2. Enlarged view of the ^{1}H NMR spectra of PHBHHx/PLA=80/20 (a) before and (b) after biodegradation.

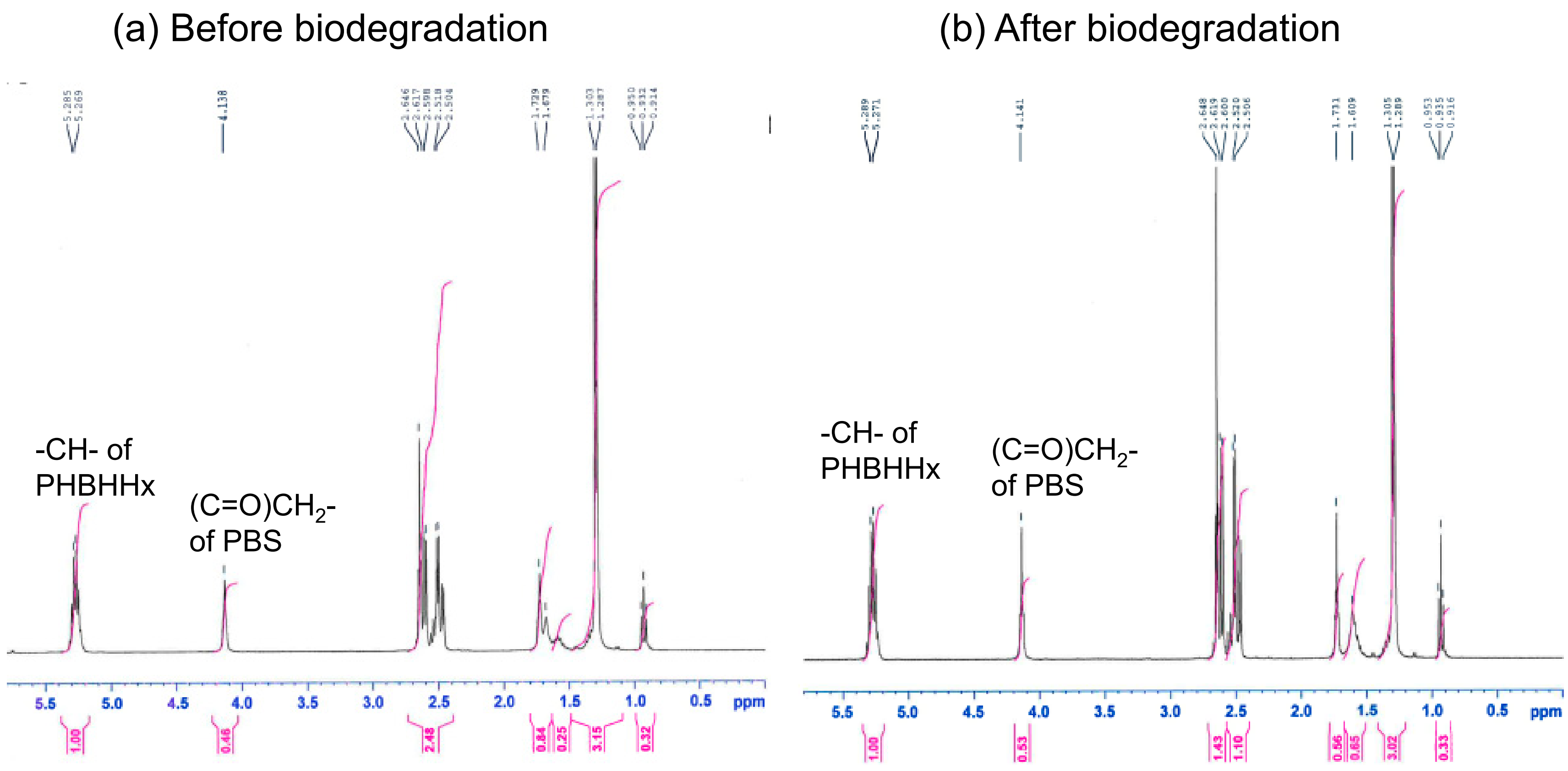

Figure S3. 1H NMR spectra of PHBHHx/PBS=80/20 (a) before and (b) after biodegradation.

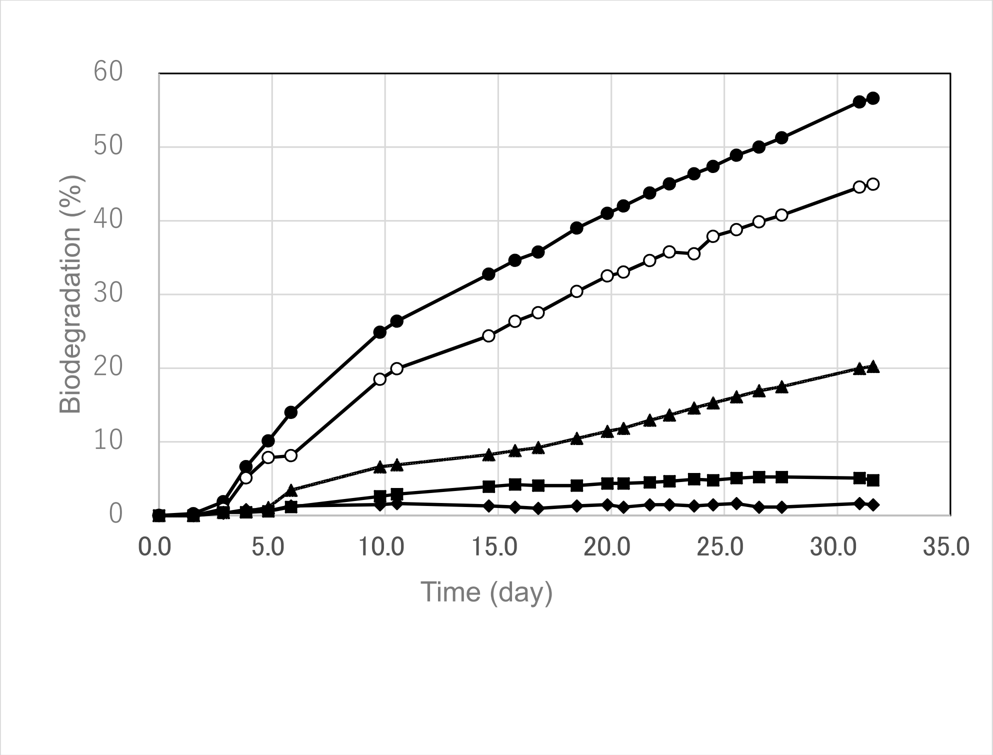

Figure S4. The biodegradation of PHBHHx and PHBHHx/PBS blends (powder shape) in seawater. ●: PHBHHx, ○: PHBHHx/PBS=80/20(wt/wt), ▲: PHBHHx/PBS=60/40(wt/wt), ■: PHBHHx/PBS=40/60(wt/wt), ◆: PBS.

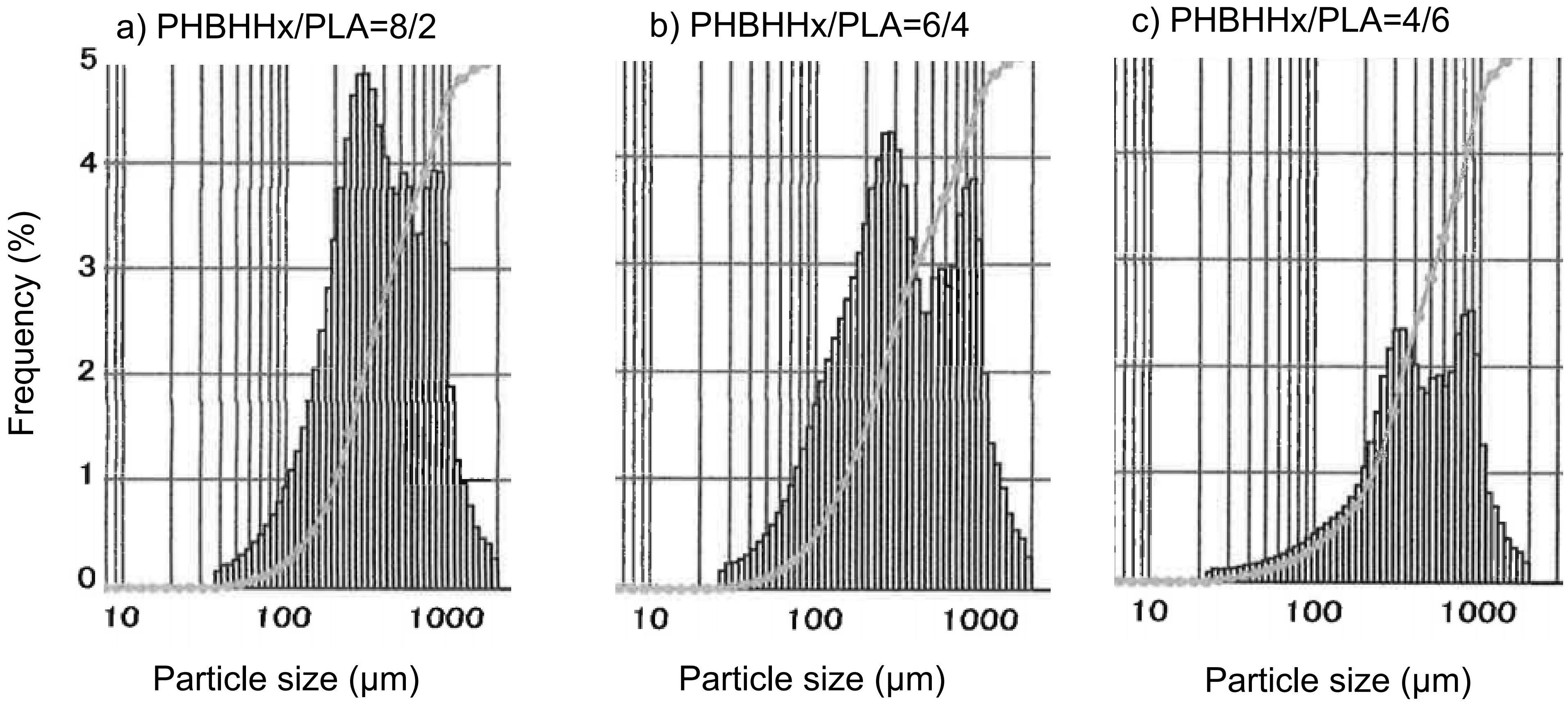

Figure S5. Particle size distribution of PHBHHx/PLA blend powders. Particle size distribution was measured using a Microtruc MT3300EXII (Nikkiso). The upper limit of measurement was 2,000 μm.
